# Quantifying the inflammatory secretome of human intermuscular adipose tissue

**DOI:** 10.14814/phy2.15424

**Published:** 2022-08-18

**Authors:** Darcy Kahn, Emily Macias, Simona Zarini, Amanda Garfield, Karin Zemski Berry, Robert Gerszten, Jonathan Schoen, Melanie Cree‐Green, Bryan C. Bergman

**Affiliations:** ^1^ Division of Endocrinology, Diabetes, and Metabolism University of Colorado Anschutz Medical Campus Aurora Colorado USA; ^2^ The Cardiovascular Research Center and Cardiology Division Massachusetts General Hospital, Harvard Medical School Boston USA; ^3^ Department of Surgery University of Colorado Anschutz Medical Campus Aurora Colorado USA; ^4^ Division of Pediatric Endocrinology University of Colorado Anschutz Medical Campus Aurora Colorado USA

**Keywords:** conditioned media, IMAT, inflammation, insulin sensitivity, paracrine signaling

## Abstract

Adipose tissue secretes an abundance of lipid and protein mediators, and this secretome is depot‐specific, with local and systemic effects on metabolic regulation. Intermuscular adipose tissue (IMAT) accumulates within the skeletal muscle compartment in obesity, and is associated with insulin resistance and metabolic disease. While the human IMAT secretome decreases insulin sensitivity in vitro, its composition is entirely unknown. The current study was conducted to investigate the composition of the human IMAT secretome, compared to that of the subcutaneous (SAT) and visceral adipose tissue (VAT) depots. IMAT, SAT, and VAT explants from individuals with obesity were used to generate conditioned media. Proteomics analysis of conditioned media was performed using multiplex proximity extension assays, and eicosanoid analysis using liquid chromatography–tandem mass spectrometry. Compared to SAT and/or VAT, IMAT secreted significantly more cytokines (IL2, IL5, IL10, IL13, IL27, FGF23, IFNγ and CSF1) and chemokines (MCP1, IL8, CCL11, CCL20, CCL25 and CCL27). Adipokines hepatocyte growth factor and resistin were secreted significantly more by IMAT than SAT or VAT. IMAT secreted significantly more eicosanoids (PGE_2,_ TXB_2_, 5‐HETE, and 12‐HETE) compared to SAT and/or VAT. In the context of obesity, IMAT is a distinct adipose tissue with a highly immunogenic and inflammatory secretome, and given its proximity to skeletal muscle, may be critical to glucose regulation and insulin resistance.

## Graphical abstract

Intermuscular adipose tissue (IMAT) is interlaced within the muscle compartment and is negatively associated with the metabolic syndrome. This is the first report of the composition of the human IMAT secretome. In obesity, the IMAT secretome is highly immunogenic, secreting a distinct combination of cytokines, chemokines, adipokines and eicosanoids that are associated with insulin sensitivity. This study highlights the potential significance of crosstalk between IMAT and skeletal muscle in the development of type 2 diabetes.

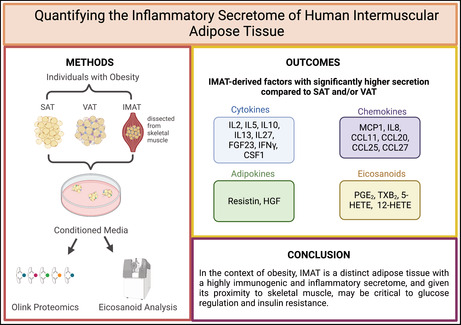

## 1 INTRODUCTION

Surrounding skeletal muscle fibers, within the fascial compartment lies an ectopic adipose tissue depot with the ability to impact skeletal muscle metabolism. While intermuscular adipose tissue (IMAT) accounts for less than 5% of total body fat, it is directly and negatively related to sarcopenia, insulin sensitivity and the metabolic syndrome (Goodpaster et al., 2000; Manini et al., 2007). The relationship between IMAT and insulin resistance was first reported in 2000 (Goodpaster et al., 2000), and has been reinforced across a wide range of individuals, by nearly every investigation of this tissue (Boettcher et al., 2009; Gallagher et al., 2005; Goodpaster et al., 2003, 2005; Miljkovic‐Gacic et al., 2008; Sinha et al., 2002; Song et al., 2004). IMAT content is higher in men compared to women (Boettcher et al., 2009), African Americans compared to Caucasians (Gallagher et al., 2005), and older compared to younger individuals (Song et al., 2004), paralleling known relationships between these populations and insulin resistance. Additionally, while intramuscular triglyceride content is known to be high in endurance trained athletes, IMAT is low in insulin sensitive athletes (Kim et al., 1985), consistent with a positive association between IMAT and insulin resistance.

Central to the association between adipose tissue and insulin resistance is its signaling and secretory properties (Hardin et al., 2008; Lehr et al., 2012). To date, we know adipose tissue secretes nearly 300 proteins, which act as endocrine, paracrine, and autocrine mediators (Lehr et al., 2012). Adipose tissues from different anatomical locations have variable secretory output, for instance, visceral adipose tissue (VAT) is known to have a significantly more pro‐inflammatory secretome than subcutaneous adipose tissue (SAT) (Fain et al., 2004). Importantly, the total body content of IMAT is similar to that of VAT, especially in women where lower VAT content is typically observed (Gallagher et al., 2005). At this time, the composition of the human IMAT secretome is unknown. However, in a previous study, our group found significant and inverse relationships between insulin sensitivity and IMAT mRNA expression of macrophage markers, as well as expression of inflammatory cytokines (Sachs et al., 2019), suggesting that IMAT may secrete inflammatory mediators known to influence tissue function. In the same study, we found that administration of IMAT and VAT conditioned media to primary muscle cell cultures resulted in significant decreases in insulin sensitivity that were not seen with SAT, and in addition, IMAT and VAT showed significantly greater lipolytic rates compared to SAT (Sachs et al., 2019). Further, genetic and epigenetic studies of porcine adipose tissue have shown that IMAT expressed genes and DNA methylation cluster with VAT, and are mainly associated with inflammatory and immune responses (Li et al., 2012; Zhou et al., 2013). Therefore, it appears that IMAT is a uniquely regulated tissue that may be functionally similar to VAT, and may have important paracrine and endocrine influence on metabolic regulation.

Due to its anatomical location, IMAT secretions are likely particularly potent for regulating insulin sensitivity in neighboring muscle. This proximity also suggests that therapeutic interventions directed at IMAT may be a powerful tool in the prevention and treatment of type 2 diabetes. While the IMAT secretome has been shown to decrease skeletal muscle insulin sensitivity in vitro (Sachs et al., 2019), its composition has not been studied in humans, therefore specific IMAT‐derived factors that impact insulin sensitivity are unknown. The goal of this study was to measure the secretome of IMAT compared to SAT and VAT in order to reveal secretory patterns that may help explain IMAT‐induced insulin resistance in skeletal muscle.

## 2 METHODS

### 2.1 Subjects

Participants in this study were recruited from two groups of individuals. Six obese IMAT donors were recruited from an ongoing clinical study, while six obese SAT/VAT donors were from a separate group of individuals undergoing bariatric surgery. Subjects gave written informed consent, and were excluded if they: had a body mass index (BMI) <30 kg/m^2^ or had liver, kidney, thyroid, or lung disease. All individuals were sedentary and engaged in planned physical activity <2 h/week. Subjects were asked to refrain from planned physical activity for 48 h before adipose tissue collection. All medications were held for 3 days prior to tissue collection. One individual in the bariatric surgery group was taking a sulfonylurea and metformin. No other individuals were taking medications known to affect insulin sensitivity. This study was approved by the Colorado Multiple Institution Review Board at the University of Colorado Anschutz Medical Campus.

### 2.2 IMAT collection

Volunteers spent the night at the Clinical Translational Research Center (CTRC) to ensure compliance with the overnight fast. In the morning after 2 h of rest, a percutaneous needle biopsy (~150 mg) was taken from midway between the greater trochanter of the femur and the patella. From within the muscle biopsy taken during this procedure, IMAT was dissected and transferred to the laboratory in low glucose (1 g/L) Dulbecco's Modified Eagle Medium (DMEM, Catalog # 10‐014‐CV; Corning) where it was prepared for the generation of conditioned media as described below.

### 2.3 Surgical tissue collection of SAT and VAT

An overnight fast was required for all patients undergoing bariatric surgery. Tissue collection commenced upon insertion of the laparoscopes. From one laparoscopic incision, superficial SAT was excised. Then, a piece of omental adipose tissue (VAT) was excised. Each piece of tissue was collected in 37°C DMEM and transported to the laboratory. SAT and VAT were dissected to remove blood and cauterized tissue, and prepared for the generation of conditioned media as described below.

### 2.4 Conditioned Media generation

The average total adipose tissue explant volume used for the generation of conditioned media was 209.5 mg for SAT, 273.8 mg for VAT and 34.8 mg for IMAT. In order to account for differences in tissue sample sizes, each piece of adipose tissue was first cut into 10–20 mg pieces, and then rinsed thoroughly in 37°C DMEM over a sterile 100 μm mesh filter. Tissue was then weighed, placed in low glucose (1 g/L) DMEM in a 20:1 ratio (μl:mg), and cultured at 37°C in 5% CO_2_ in sterile, polystyrene culture dishes (Corning). Due to the cytotoxicity that is observed during the first hour of incubation which is the result of tissue processing, after 1 h, DMEM was discarded to ensure conditioned media was not contaminated by factors released from cellular damage and tissue handling. Fresh low glucose (1 g/L) DMEM was replaced in a 20:1 ratio (μl:mg) and cultured at 37°C in 5% CO_2_ for 24 h.

### 2.5 Olink analysis

Adipose tissue conditioned media was evaluated by the Olink Target 96 platform that quantified 1072 proteins (Olink Bioscience AB), using multiplex proximity extension assay panels, as previously described (Assarsson et al., 2014). Quantification of secreted proteins was normalized to tissue sample dry weight. The following Olink panels were used in the analysis: Cardiometabolic, Cardiovascular II, Cardiovascular III, Cell Regulation, Development, Immune Response, Inflammation, Metabolism, Neuro Exploratory, Neurology, Oncology II, Oncology III, and Organ Damage. Full lists of proteins quantified in each panel can be found at: www.olink.com/products‐services/target.

### 2.6 Eicosanoid analysis

Eicosanoid analysis was performed on adipose tissue conditioned media by liquid chromatography–tandem mass spectrometry (LC–MS/MS) as previously described (Zarini et al., 2009). Briefly, after addition of a deuterated internal standard mixture, SAT, VAT and IMAT conditioned media samples were added to MeOH (1:2), centrifuged, and then extracted using a solid phase extraction cartridge (Strata‐X 33 μm Polymeric Reversed Phase; Phenomenex). Metabolites were eluted, dried down, reconstituted, injected and separated on an HPLC column (Gemini C18, 150 × 2 mm, 5 μm; Phenomenex) directly interfaced into the electrospray source of a triple quadrupole mass spectrometer (4000 QTRAP; Sciex). Quantitation was performed using standard isotope dilution curves, as previously described (Zarini et al., 2009), and normalized to tissue dry weight.

### 2.7 Statistical analysis

Data are presented as mean ± SEM. Internal plate standardization and quality control for the proteomics were performed by Olink, exporting normalized protein expression values. Differences in normally distributed data between groups were analyzed using a one‐way analysis of variance (ANOVA; SPSS). Non‐normally distributed data were log‐transformed before analysis using a one‐way ANOVA. When significant differences were detected, individual means were compared using Student's *t*‐tests to determine differences between groups.

## 3 RESULTS

### 3.1 Demographics

The demographics of individuals undergoing bariatric surgery, as well as participants in the metabolic study are shown in Table 1. The mean age, fasting glucose, and HbA1c were not different between groups. However, the mean BMI of the bariatric patients was significantly greater than the IMAT subjects (*p* < 0.05). Considering VAT and SAT are likely to be more adverse in this population with a greater degree of obesity, it is possible that the differences between IMAT and VAT/SAT may be under‐represented in the current comparisons.

**TABLE 1:** Study participant demographics

Variable	IMAT donors	SAT/VAT donors
*N* (W/M)	6 (4/2)	6 (5/1)
Age (years)	43.5 ± 1.8	40.5 ± 2.6
BMI (kg/m^2^)	35.7 ± 1.7	47.4 ± 3.5*
Fasting glucose (mg/dl)	86.0 ± 3.5	82.3 ± 5.5
A1c (%)	5.8 ± 0.1	5.7 ± 0.1

*Note*: Values are means ± SEM.

Abbreviations: BMI, body mass index; IMAT, intermuscular adipose tissue; SAT, subcutaneous adipose tissue; VAT, visceral adipose tissue.

* Significantly different than IMAT donors, *p* < 0.05.

### 3.2 Basal lipolytic rates of SAT, VAT, and IMAT

SAT‐, VAT‐, and IMAT‐conditioned media were lipid extracted and analyzed for free fatty acids (FFA) using lipidomics techniques, with FFA release rates normalized to dry tissue weight. VAT and IMAT have similar lipolytic rates, which were both significantly greater than SAT (SAT: 0.30 ± 0.11, VAT: 1.24 ± 0.30, IMAT: 1.26 ± 0.10 nmol FFA released/hr/mg dry weight).

### 3.3 Cytokine secretion

**FIGURE 1:**
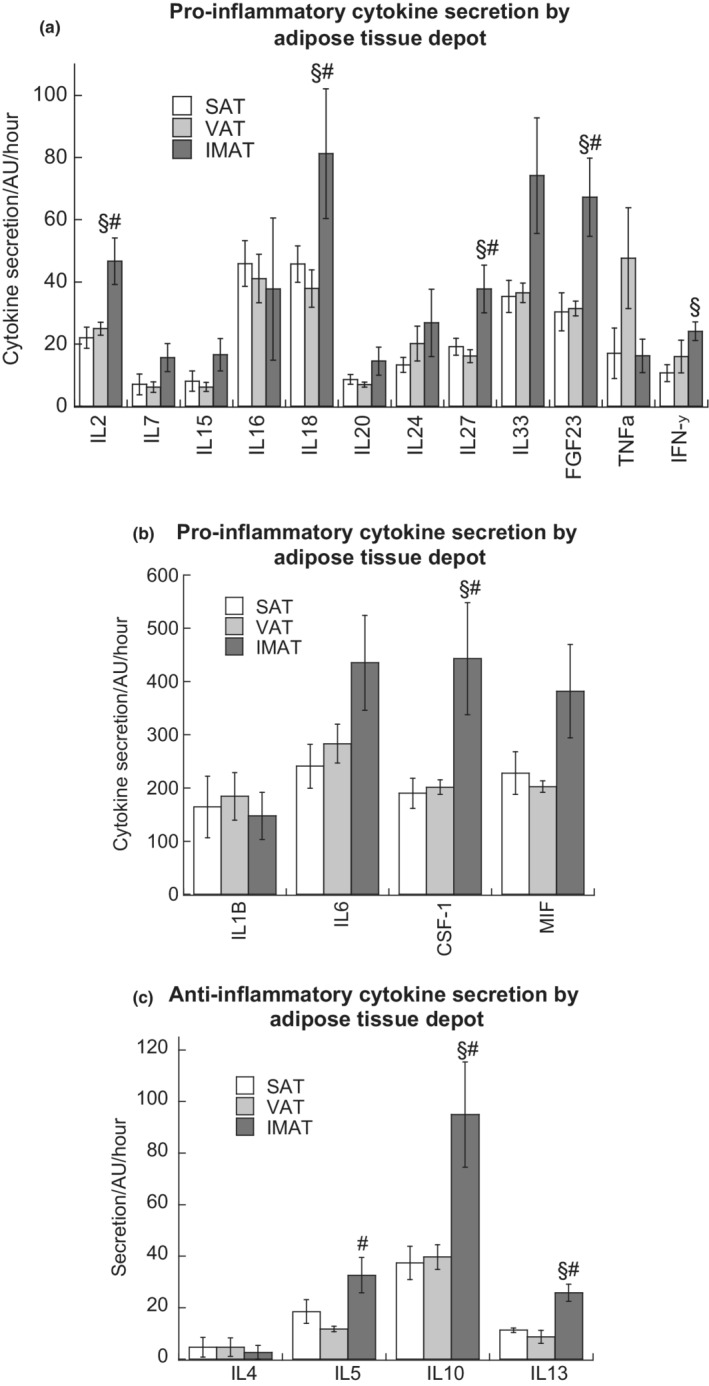
Secretion of low (a) and high (b) abundance pro‐inflammatory cytokines, as well as anti‐inflammatory cytokines (c) from SAT, VAT, and IMAT. Values are means ± SEM. ^§^Significantly different than SAT, ^#^Significantly different that VAT, *p* < 0.05. IMAT, intermuscular adipose tissue; SAT, subcutaneous adipose tissue; VAT, visceral adipose tissue

IMAT secretion of pro‐inflammatory cytokines IL2, IL18, IL27, FGF23, and CSF1 were significantly greater than SAT and VAT (*p* < 0.05) and interferon gamma secretion from IMAT was significantly greater compared to SAT (Figure 1a,b). IMAT secretion of anti‐inflammatory cytokines IL10 and IL13 was significantly greater than SAT and VAT (*p* < 0.05), and IL5 secretion from IMAT was significantly greater compared to VAT (*p* < 0.05, Figure 1c). Thus, IMAT production of inflammatory mediators is balanced, with higher secretion of both pro and anti‐inflammatory cytokines.

### 3.4 Chemokine secretion

**FIGURE 2:**
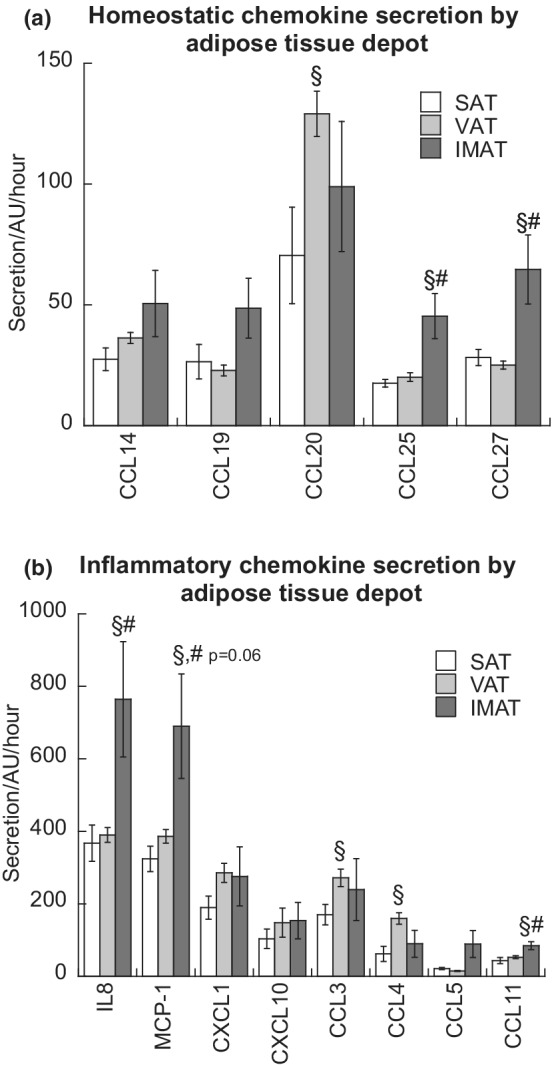
Homeostatic chemokine (a) and inflammatory chemokine (b) secretion from SAT, VAT, and IMAT. Values are means ± SEM. ^§^Significantly different than SAT, ^#^Significantly different that VAT, *p* < 0.05. IMAT, intermuscular adipose tissue; SAT, subcutaneous adipose tissue; VAT, visceral adipose tissue

While some chemokines are expressed constitutively in specific tissues or cells (‘homeostatic chemokines’), ‘inflammatory chemokines’ are induced during inflammation and play a critical role in recruiting cells involved in immune and inflammatory response to adipose tissue (Zlotnik et al., 2011). IMAT secretion of the homeostatic chemokines CCL25 and CCL27, as well as the inflammatory chemokines CCL11 and IL8 was significantly higher compared to SAT and VAT (*p* < 0.05, Figure 2a,b). IMAT secretion of MCP1 was higher compared to SAT (*p* < 0.05), and compared to VAT, the difference approached significant levels (*p* = 0.06, Figure 2b). VAT secretion of CCL20 was significantly greater than SAT (*p* < 0.05, Figure 2a).

### 3.5 Adipokine secretion

**FIGURE 3:**
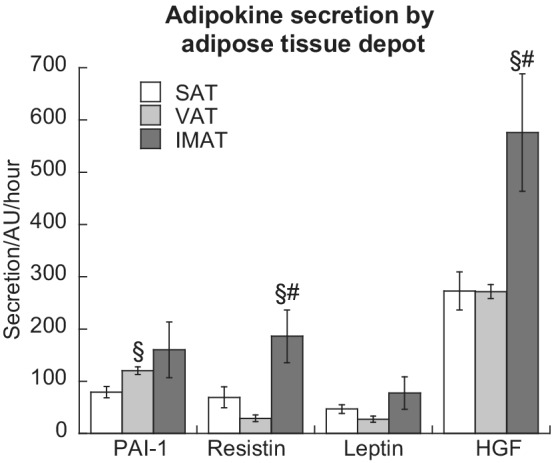
Adipokine secretion from SAT, VAT, and IMAT. Values are means ± SEM. ^§^Significantly different than SAT, ^#^Significantly different that VAT, *p* < 0.05. IMAT, intermuscular adipose tissue; SAT, subcutaneous adipose tissue; VAT, visceral adipose tissue

We then examined the secretion of adipokines from IMAT, VAT, and SAT depots using proteomic analysis of conditioned media generated from the three different adipose tissues. Plasminogen activator inhibitor 1 secretion was significantly greater in VAT compared to SAT (*p* < 0.05, Figure 3). Resistin and hepatocyte growth factor (HGF) secretion was significantly greater in IMAT compared to SAT and VAT (*p* < 0.05) while there were no differences in leptin secretion. Additionally, there were no differences in adiponectin secretion between depots (data not shown).

Composite figures integrating all cytokines, chemokines, and adipokines for each adipose tissue depot can be found in Figure S1a–c (https://figshare.com/s/9fcae399c9ade5fc821a). A complete list of proteins quantified by Olink proteomics in the SAT, VAT, and IMAT secretome can be found in Supplemental Table 1 (https://figshare.com/s/5c176367336f811efb61).

### 3.6 Eicosanoid secretion

**FIGURE 4:**
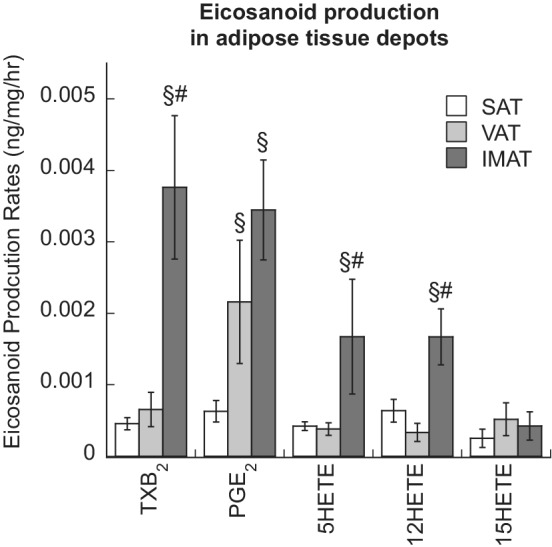
Eicosanoid secretion from subcutaneous (SAT), visceral (VAT), and intermuscular adipose tissue (IMAT). Values are means ± SEM. ^§^Significantly different than SAT, ^#^Significantly different that VAT, *p* < 0.05.

Obesity is associated with increased production of bioactive lipid mediators, such as arachidonic acid‐derived eicosanoids, that regulate pro‐inflammatory signaling in a variety of different tissues (Pickens et al., 2017). Therefore, we were interested in quantifying eicosanoid production and secretion by IMAT, compared to VAT and SAT. We found that IMAT secretes more thromboxane B_2_ (TXB_2_), 5‐hydroxyeicosatetraenoic acid (5‐HETE), and 12‐hydroxyeicosatetraenoic acid (12‐HETE) than both SAT and VAT, while IMAT and VAT secrete similar amounts of pro‐inflammatory prostaglandin E_2_ (PGE_2_) compared to SAT (*p* < 0.05, Figure 4). There were no differences in 15‐hydroxyeicosatetraenoic acid (15‐HETE) between groups.

Receptor expression for IMAT‐derived cytokines, chemokines, adipokines, and eicosanoids with significantly higher secretion compared to SAT and VAT in skeletal muscle is described in Table 2.

**TABLE 2:** Receptor expression for IMAT‐derived factors with significantly higher secretion compared to SAT and VAT

IMAT‐secreted factors	Uniprot ID	Receptor/s	Receptor‐expressing cell type in skeletal muscle
IL2	P60568	IL‐2R α/β/γ	Skeletal myotubes (Yoshida et al., 2022), T‐cells, macrophages
IL18	Q14116	IL‐18Rα/β	Endothelial cells, macrophages, neutrophils
IL27	Q8NEV9	IL‐27Rα, GP130	Endothelial cells, macrophages, neutrophils
FGF23	Q9GZV9	FGFR1, Klotho	Satellite cells (Kästner et al., 2000), skeletal myotubes (Avin et al., 2018), endothelial cells fibroblasts, macrophages
CSF1	P09603	CSF1R	Endothelial cells, fibroblasts, macrophages
IL10	P22301	IL‐10Rα/β	Skeletal myoblasts (Strle et al., 2007), endothelial cells, fibroblasts, macrophages, neutrophils
IL13	P35225	IL‐13Rα1, IL‐4Rα	Skeletal myotubes (Prokopchuk et al., 2007), endothelial cells, macrophages, neutrophils
IL8	P10145	CXCR1, CXCR2	Skeletal myotubes (Sell et al., 2006), neutrophils
CCL11	P51671	CCR2, CCR3, CCR5	Skeletal myotubes (Sell et al., 2006), macrophages
MCP1	Q6UZ82	CCR2, CCR4	Skeletal myotubes (Sell et al., 2006), macrophages
CCL25	O15444	CCR9	Not detected
CCL27	Q9Y4X3	CCR10	Skeletal myotubes (Sell et al., 2006), fibroblasts
Resistin	Q9HD89	TLR4, CAP1	Skeletal myotubes (Radin et al., 2008), endothelial cells, macrophages, neutrophils
HGF	P14210	c‐Met	Satellite cells (Kästner et al., 2000), skeletal myotubes (Gal‐Levi et al., 1998), macrophages
TXB_2_	n/a	TPα/β	Endothelial cells, fibroblasts
5‐HETE	n/a	OXER1	Fibroblasts, macrophages
12‐HETE	n/a	GPR31	Macrophages

*Note*: This table includes well‐known receptors and receptor‐expressing cell types in skeletal muscle at the time of publication, and is not meant to be an exhaustive list of all potential ligand/receptor interactions or all cell types that may express the receptors. To provide context for the in vitro and in vivo relationship between intermuscular adipose tissue and skeletal muscle insulin sensitivity references are included for those factors with reported expression on skeletal myotubes.

## 4 DISCUSSION

Secretions from adipose tissue are able to modulate critical metabolic processes through endocrine signaling interactions, yet they also have local, depot‐specific impact on neighboring tissues. The paracrine relationship between IMAT and skeletal muscle is of particular relevance to the dysregulation of glucose homeostasis and insulin resistance that is observed in metabolic disease, and while we have previously shown that IMAT conditioned media decreases insulin sensitivity in skeletal muscle cells in vitro (Sachs et al., 2019), little is known about the composition of the IMAT secretome. In this study we found that IMAT is highly immunogenic, secreting significantly higher levels of certain cytokines and chemokines than other adipose tissue depots. Additionally, IMAT is a significant contributor of bioactive lipids and adipokines that regulate metabolism in skeletal muscle. These data suggest that skeletal muscle receptor signaling by IMAT‐derived inflammatory protein and lipid mediators may help explain the negative effect of IMAT‐conditioned media on insulin sensitivity in vitro (Table 2) (Sachs et al., 2019), and provides insight into the negative clinical relationship between IMAT content and metabolic risk in humans (Goodpaster et al., 2005).

In obesity, a proinflammatory shift in adipose tissue immune population is observed (Lu et al., 2019; Rausch et al., 2008), which is in part due to increased expression and secretion of inflammatory chemokines leading to macrophage and T cell chemotaxis (Rehman & Akash, 2016). While chemokine‐induced accumulation of proinflammatory immune cells has been reported in VAT (Curat et al., 2006), this phenomenon has not previously been described in IMAT. In the current study, we observed higher levels of IMAT secretion of a variety of chemokines which have important and differing roles in the recruitment of immune cells and the maintenance of adipose tissue inflammation.

Monocyte chemoattractant protein 1 (MCP1) is an abundant and ubiquitous chemokine that has been studied extensively in obesity and metabolic disease (Catalan et al., 2007). Increased adipose tissue expression and secretion of MCP1, has been reported in individuals with obesity (Bremer et al., 2011; Bruun et al., 2005), with increased levels in visceral compared to subcutaneous adipose tissue compartments (Harman‐Boehm et al., 2007). In the current study, higher MCP1 secretion, partnered with elevated secretion of a variety of pro‐inflammatory cytokines from IMAT, corroborates previous reports from our group that describe a positive association between macrophage markers/cytokine mRNA expression and insulin resistance (Sachs et al., 2019).

In addition to its chemoattractant capabilities, MCP1 has been shown to regulate other physiological processes, including angiogenesis and insulin sensitivity (Salcedo et al., 2000; Sartipy & Loskutoff, 2003). Obese mice lacking the MCP1 receptor CCR2 have increased adiponectin expression, ameliorated hepatic steatosis, and improved systemic glucose homeostasis (Weisberg et al., 2006). Importantly, MCP1 is a potent regulator of skeletal muscle inflammation and insulin sensitivity. It has been shown that human skeletal muscle cells are highly sensitive toward MCP1, which impairs insulin signaling and glucose uptake at concentrations 10 times less than what is typically found in circulation (Sell et al., 2006). Furthermore, muscle‐specific overexpression of MCP1 leads to increased expression of macrophage markers, chemokines, and cytokines, as well as local disruption of insulin signaling in mice (Patsouris et al., 2014). Elevated release of MCP1 by IMAT into the extracellular space shared with skeletal muscle could be an important contributor to skeletal muscle inflammation and insulin resistance in obesity.

IMAT secreted more CC motif chemokine ligand 11 (CCL11) and IL5, which are both inducers of eosinophil migration (Bolus et al., 2018; Ponath et al., 1996). In adipose tissue, eosinophils modulate the immune microenvironment, supporting the accumulation of M2‐like macrophages which generate anti‐inflammatory cytokines including IL4, IL10, IL13, and TGFβ (Lee et al., 2018; Ruytinx et al., 2018; Wu et al., 2011). Mirroring this immune process, greater secretion of IL10 and IL13 was observed from IMAT compared to the other two adipose depots. Higher secretion of anti‐inflammatory mediators from IMAT is in contrast to previous findings by our group and others which highlight the negative association between IMAT and local as well as systemic inflammation and insulin resistance (Boettcher et al., 2009; Sachs et al., 2019). However, there have been many reports of increased levels of anti‐inflammatory cytokines in obesity and type 2 diabetes that are similarly conflicting to typical associations between inflammation and metabolic dysfunction (Binisor et al., 2016; Esposito et al., 2003; Martinez‐Reyes et al., 2018). One novel and compelling explanation for elevated anti‐inflammatory cytokines in type 2 diabetes is the development of “cytokine resistance” in which anti‐inflammatory mediators such as IL10 and IL4 lose the ability to inhibit pro‐inflammatory cytokine synthesis and secretion due to disrupted signaling in a state of chronic inflammation and insulin resistance (Barry et al., 2016; O'Connor et al., 2007). The association between IMAT and insulin resistance previously reported, in conjunction with the higher secretion of both pro‐ and anti‐inflammatory cytokines from IMAT observed in this study, may suggest the development of cytokine resistance associated with chronically inflamed adipose tissue in obesity. This could lead to an unbalanced inflammatory environment within the skeletal muscle compartment and could contribute to insulin resistance.

IMAT had elevated secretion of IL8, which is a member of the CXC family of chemokines that promotes migration of neutrophils and leukocytes (Fujiwara et al., 2002). In addition to its role in inflammatory response, IL8 is known to be a potent inducer of angiogenesis with expression in muscle induced by exercise (Koch et al., 1992; Nielsen & Pedersen, 2007). However, IL8 expression is positively associated with insulin resistance (Zozulinska et al., 1999). There is evidence to suggest that the effects of IL8 are hormetic, with low doses resulting in stimulatory outcomes, while high doses can exert inhibitory effects (Amir Levy et al., 2015). Interestingly, exercise‐induced increase in IL8 is observed in skeletal muscle, but not systemically, and therefore IL8 is thought to have a very local effect (Chan et al., 2004). In addition to increased IL8 production from insulin resistant muscle itself, the high levels of IL8 released from IMAT in obesity may result in elevated local levels of IL8, which is associated with reduced substrate delivery, in addition to inflammation (Amir Levy et al., 2015). This highlights the potential significance of local crosstalk between skeletal muscle and IMAT in obesity and type 2 diabetes.

The secretion of chemokines and inflammatory cytokines from IMAT is suggestive of increased populations of macrophages, which have been shown to be the predominant source of the adipokine resistin in adipose tissue (Curat et al., 2006). Indeed, in this study, resistin was secreted in abundance from IMAT. The mechanistic impact of resistin on skeletal muscle has not been fully elucidated, however studies have found that resistin causes impaired IRS1 activation, GLUT4 translocation, and glucose uptake in rat myotubes in vitro (Fan et al., 2007; Palanivel et al., 2006). Additionally, resistin is associated with the stimulation of lipolysis (Ort et al., 2005; Qatanani et al., 2009), which, combined with increased interstitial FFA may contribute to skeletal muscle inflammation and insulin resistance. Thus, the potential local effect of IMAT‐derived resistin on skeletal muscle function highlights the negative consequences of IMAT accumulation on skeletal muscle that can contribute to the development of insulin resistance.

Hepatocyte growth factor is an IMAT‐secreted adipokine that promotes epithelial cell proliferation, motility, and morphogenesis (Bell et al., 2006; Fain et al., 2004). HGF is positively associated with BMI and insulin resistance (Rajpathak et al., 2010; Tsukagawa et al., 2013), and decreases after weight loss (Bell et al., 2006). Despite links to obesity and insulin resistance, HGF promotes increased glucose uptake in pancreatic islets, adipocytes, and myotubes in vitro (Bertola et al., 2007; Garcia‐Ocana et al., 2001; Perdomo et al., 2008). Our data extend the literature to show that IMAT secretes more HGF than SAT and VAT, and therefore may antagonize the negative effects of the IMAT secretome on insulin sensitivity in skeletal muscle.

Secretion of eicosanoids such as prostaglandins and thromboxanes may contribute to the active inflammatory state of IMAT in obesity. These signaling lipids act in an autocrine and paracrine manner to promote inflammation and insulin resistance via enhanced macrophage chemotaxis (Chan et al., 2016; Hata & Breyer, 2004). Circulating levels of TXB_2_, the stable metabolite of thromboxane A_2_, is greater in insulin resistant individuals with obesity, as well as type 1 and 2 diabetes, and has been shown to cause whole body insulin resistance, as well as adipose tissue fibrosis in rodents (Graziani et al., 2011; Lei et al., 2015; Pulcinelli et al., 2009). Evidence of systemic as well as local effects of prostaglandins and thromboxanes, in addition to higher secretion of PGE_2_ and TXB_2_ by IMAT observed in the current study, suggests that these eicosanoids may have the potential to negatively influence muscle insulin sensitivity in humans.

Another class of eicosanoids linked to systemic insulin resistance and adipose tissue inflammation are the hydroxyeicosatetranoic acids (HETEs). 12‐HETE promotes adipose tissue macrophage infiltration and expression of MCP1, IL6, and TNFα through autocrine and paracrine signaling (Chakrabarti et al., 2009; Law et al., 2010; Nunemaker et al., 2008). 5‐HETE, as well as the oxidized form 5‐oxo‐ETE, have been shown to be potent promoters of neutrophil, monocyte and eosinophil chemotaxis and therefore may contribute to skeletal muscle inflammation (Kern et al., 2018; Powell & Rokach, 2005). Combined, IMAT secretion of HETEs may contribute to a pro‐inflammatory shift in IMAT, impacting the micro‐environment within the skeletal muscle compartment.

We have previously shown that IMAT conditioned media causes decreased insulin sensitivity in vitro (Sachs et al., 2019), and while this study advances our understanding of the relationship between IMAT secretions and muscle insulin sensitivity, there is still much that is unknown. There are no studies addressing whether the IMAT secretome is altered in obesity and/or type 2 diabetes. Moreover, investigations into differences in the IMAT secretome based on ethnicity, race, sex, or age do not yet exist, nor is it known if lifestyle modifications such as weight loss or exercise training impact IMAT paracrine signaling. Understanding the malleability of the IMAT secretome is critical to the development of interventions to prevent IMAT‐induced muscle metabolic dysfunction.

In summary, the current work has shown that in obesity, IMAT is a distinct secretory tissue with a highly inflammatory secretome. This unique composition of cytokines, chemokines, adipokines, and eicosanoids are anatomically positioned to directly impact skeletal muscle inflammation and insulin sensitivity. The findings from this study expand what is known about the adipose tissue secretome, and highlight the potential significance of local crosstalk between IMAT and skeletal muscle in the development of insulin resistance and type 2 diabetes.

## AUTHOR CONTRIBUTIONS

Bryan C. Bergman is the study sponsor/funder of this work and, as such, had full access to all the data in the study and takes responsibility for the integrity of the data and the accuracy of the data analysis. Darcy Kahn performed subject testing, sample analysis, helped interpret the data and wrote the manuscript, Emily Macias performed subject testing, sample analysis, and edited the manuscript, Simona Zarini helped perform subject testing and edited the manuscript, Amanda Garfield helped perform subject testing and edited the manuscript, Karin Zemski Berry help perform eicosanoid sample analysis, interpreted data, and edited the manuscript, Robert Gerszten performed Olink analysis on the samples and edited the manuscript, Jonathan Schoen provided medical oversight, helped perform biopsies, and edited the manuscript, Melanie Cree‐Green provided medical oversight, helped perform biopsies, and helped interpret the data and write the manuscript, Bryan C. Bergman designed the study, performed subject testing, analyzed and interpreted data, and helped write the manuscript. Graphical Abstract created with BioRender.com.

## FUNDING INFORMATION

This work was partially supported by the National Center for Research Resources grant RR‐00036, National Institute of Diabetes and Digestive and Kidney Diseases (NIDDK) grants to Bryan C. Bergman (R01DK111559 and R01DK118149), National Institute of Diabetes and Digestive and Kidney Diseases (NIDDK) grants to Darcy E Kahn (F31DK126393) and the Colorado Nutrition Obesity Research Center grant P30DK048520.

## AUTHORS' RELATIONSHIPS AND ACTIVITIES

The authors declare that there are no relationships or activities that may bias, or be perceived to bias, this work.

## CONFLICT OF INTEREST

The authors have no confict of interest to disclose.

## ETHICS STATEMENT

This study was approved by the Colorado Multiple Institution Review Board at the University of Colorado Anschutz Medical Campus.

## Supporting information


Appendix S1
