# Supplementary figures and images for: Quantifying the inflammatory secretome of human intermuscular adipose tissue
Source: Physiol Rep. 2022 Aug 18;10(16):e15424. doi: 10.14814/phy2.15424 (PMC9387112; doi:10.14814/phy2.15424)

# Cytokine and adipokine secretion in SAT

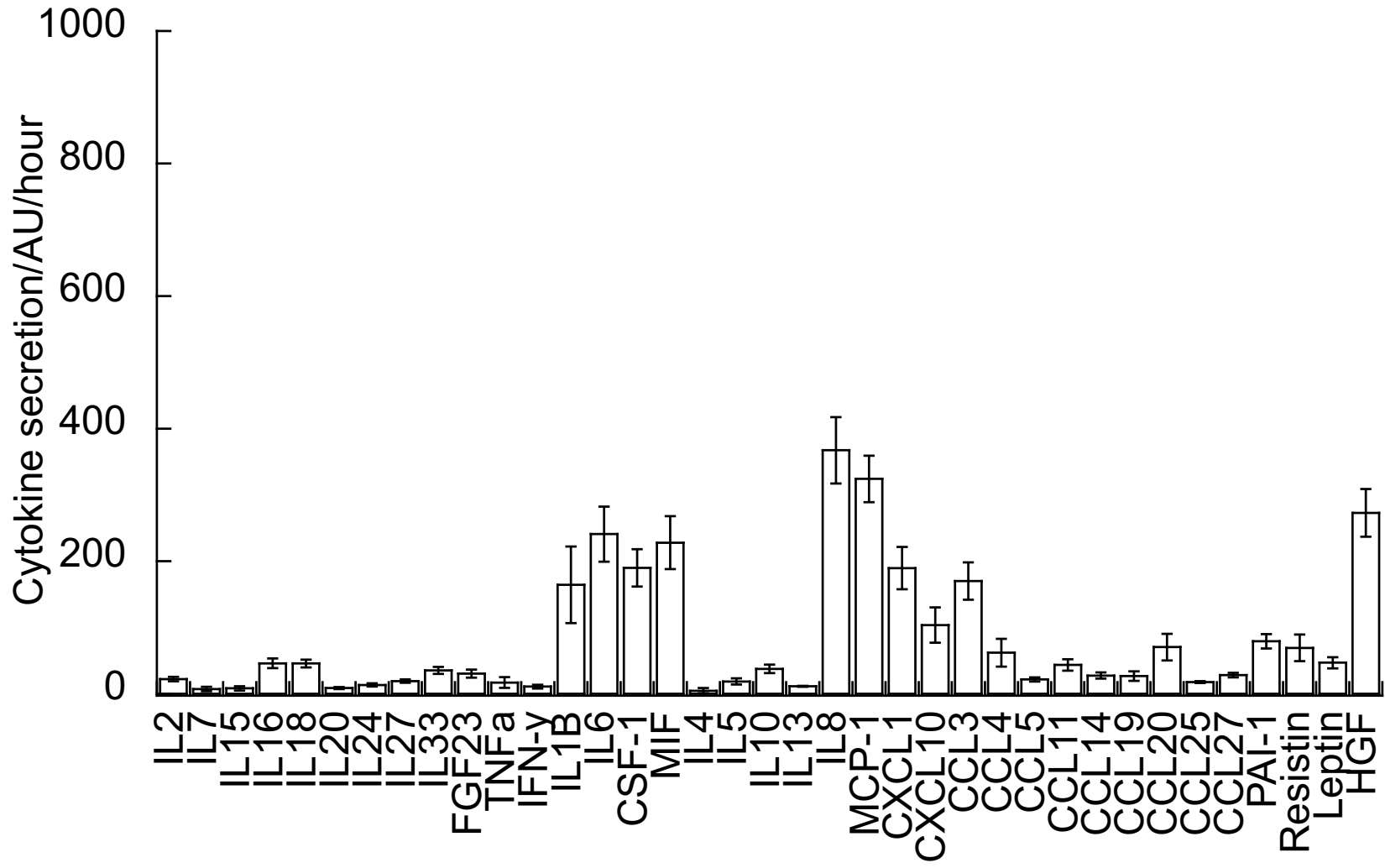

Supplement: Supplementary file 1 — Appendix S1 [file PHY2-10-e15424-s001.zip › phy215424-sup-0001-FigureS1A.pdf]

# Cytokine and adipokine secretion in VAT

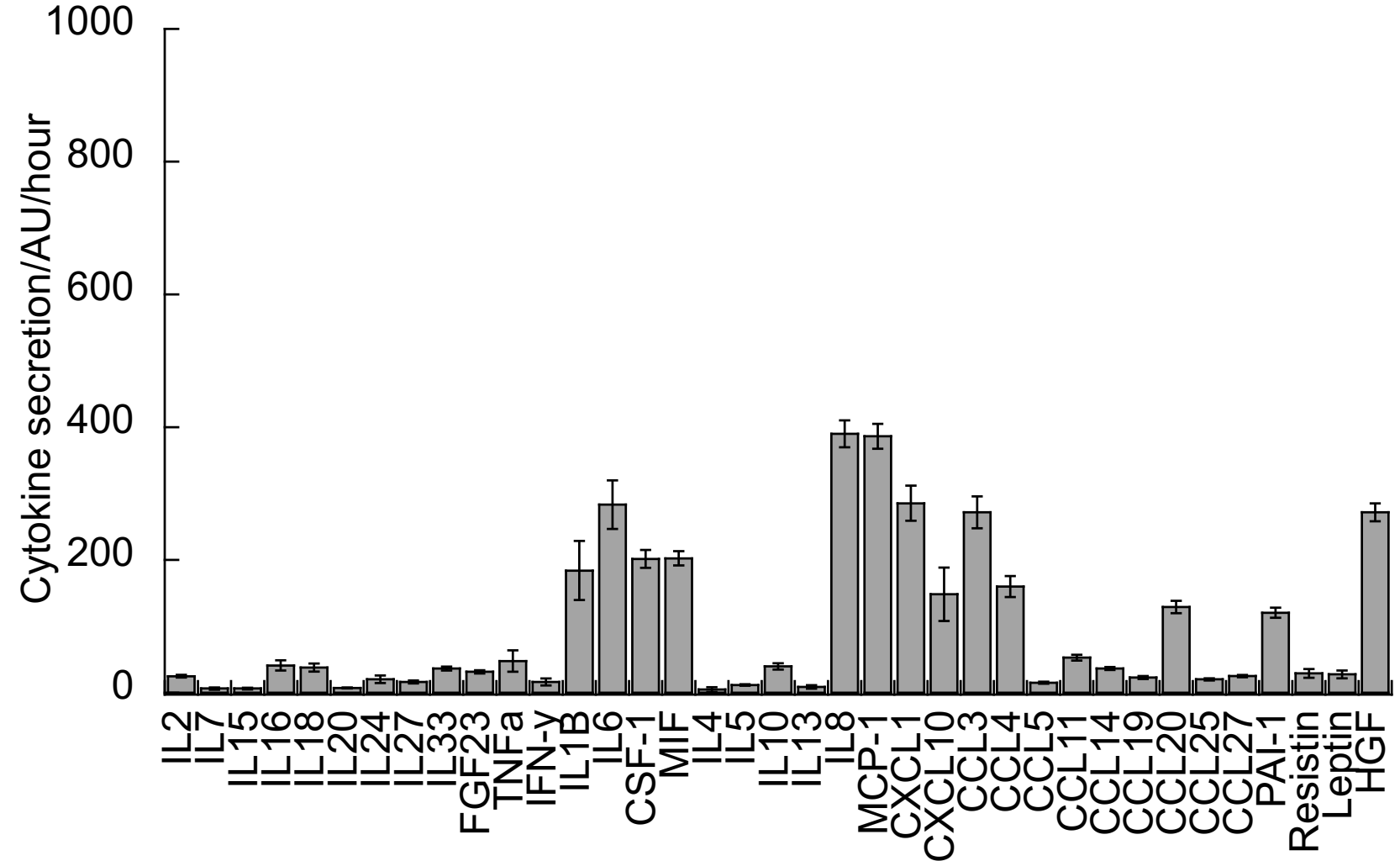

Supplement: Supplementary file 1 — Appendix S1 [file PHY2-10-e15424-s001.zip › phy215424-sup-0002-FigureS1B.pdf]

# Cytokine and adipokine secretion in IMAT

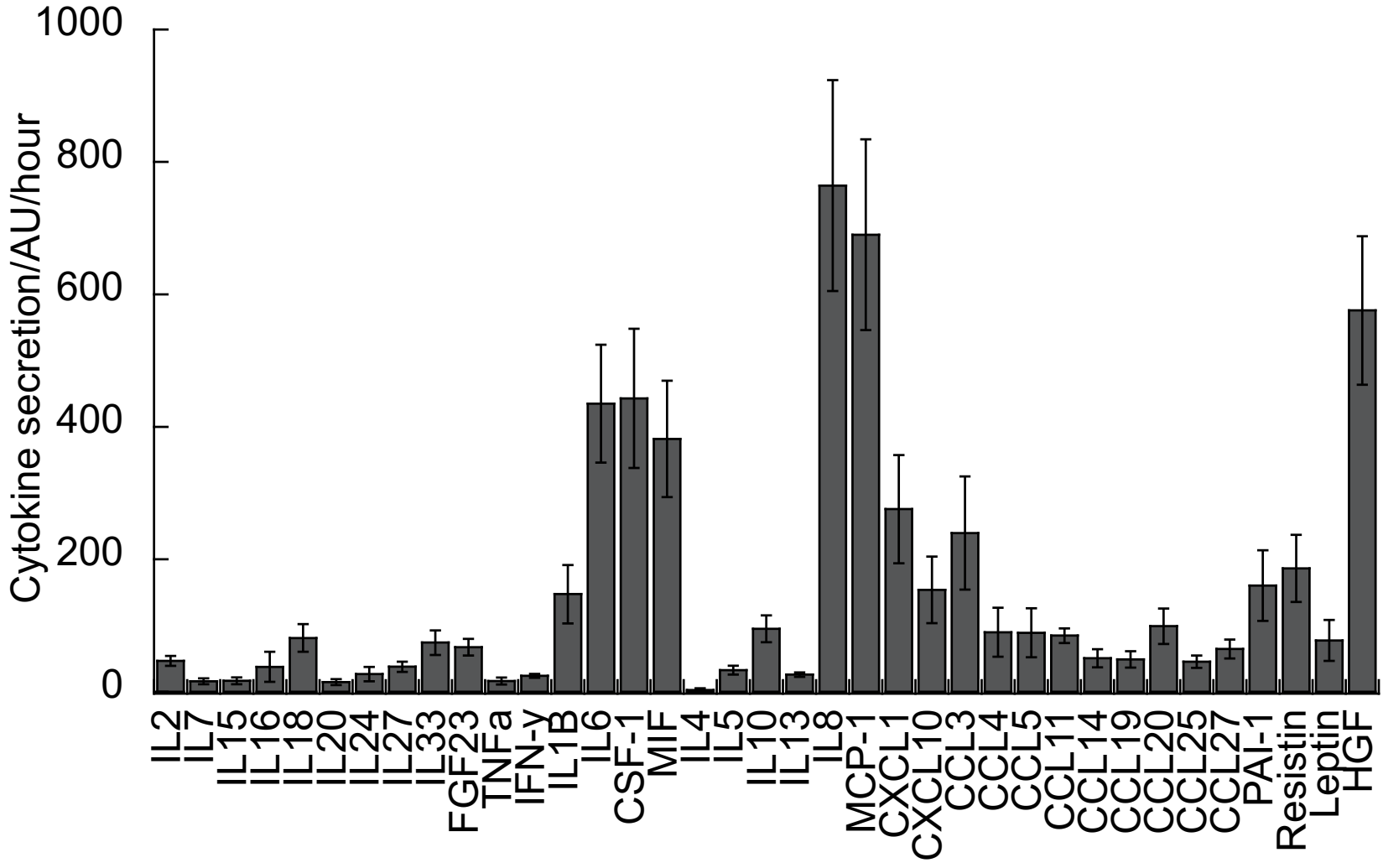

Supplement: Supplementary file 1 — Appendix S1 [file PHY2-10-e15424-s001.zip › phy215424-sup-0003-FigureS1C.pdf]
